# *Engineering* creativity: Prior experience modulates electrophysiological responses to novel metaphors

**DOI:** 10.1111/psyp.13630

**Published:** 2020-07-16

**Authors:** Rafal Jończyk, Gül E. Kremer, Zahed Siddique, Janet G. van Hell

**Affiliations:** ^1^ Faculty of English Adam Mickiewicz University Poznań Poland; ^2^ Department of Psychology Pennsylvania State University State College PA USA; ^3^ Department of Industrial and Manufacturing Systems Engineering Iowa State University Ames IA USA; ^4^ The School of Aerospace and Mechanical Engineering University of Oklahoma Norman OK USA

**Keywords:** ERPs, Creativity, N400, Metaphor, Figurative language, Prior Knowledge

## Abstract

Novel metaphorical language use exemplifies human creativity through production and comprehension of meaningful linguistic expressions that may have never been heard before. Available electrophysiological research demonstrates, however, that novel metaphor comprehension is cognitively costly, as it requires integrating information from distantly related concepts. Herein, we investigate if such cognitive cost may be reduced as a factor of prior domain knowledge. To this end, we asked engineering and nonengineering students to read for comprehension literal, novel metaphorical, and anomalous sentences related to engineering or general knowledge, while undergoing EEG recording. Upon reading each sentence, participants were asked to judge whether or not the sentence was original in meaning (*novelty* judgment) and whether or not it made sense (*sensicality* judgment). When collapsed across groups, our findings demonstrate a gradual N400 modulation with N400 being maximal in response to anomalous, followed by metaphorical, and literal sentences. Between‐group comparisons revealed a mirror effect on the N400 to novel metaphorical sentences, with attenuated N400 in engineers and enhanced N400 in non‐engineers. Critically, planned comparisons demonstrated reduced N400 amplitudes to engineering novel metaphors in engineers relative to non‐engineers, pointing to an effect of prior knowledge on metaphor processing. This reduction, however, was observed in the absence of a sentence type × knowledge × group interaction. Altogether, our study provides novel evidence suggesting that prior domain knowledge may have a direct impact on creative language comprehension.

## Abstract 2

This study provides novel evidence that prior knowledge facilitates interpretation of novel metaphorical sentences, an index of linguistic creativity. As such, this study contributes to, and bridges, the fields of figurative language processing and creativity research.

## 1 INTRODUCTION

Creativity is a unique human trait that has been broadly defined as the ability to produce work that is both novel (i.e., original, unusual) and appropriate (useful, relevant to the task at hand; Sternberg & Lubart, 1996). Language is a prime illustration of the human creative potential. Not only are we able to communicate an infinite number of meaningful linguistic expressions, some of which may not have been heard before, we can also decipher new thoughts and ideas that are being communicated to us. In essence, humans are not only creative in how they produce language but, equally so, in how they comprehend language.

Arguably, figurative language use such as metaphors provides the most powerful tool to create new meaning (for a discussion, see Cacciari & Glucksberg, 1994). We view metaphors in terms of conceptual processes rather than as strictly linguistic entities, in line with Cacciari and Glucksberg (1994). According to them (Cacciari & Glucksberg, 1994, p. 448), a metaphor is “any linguistic expression that is intended and/or recognized as a metaphor by a speaker or listener (writer or reader)”. The processing of novel metaphorical expressions, in particular, may provide a unique insight into linguistic creativity. Indeed, novel metaphorical language use signifies the human ability to produce new and meaningful linguistic expressions that enrich communication and have the potential to implant new thoughts and ideas in the listeners' mind. This, however, may come at a price of increased comprehension costs in readers or listeners who may need more time and effort to integrate information from semantically distant concepts (Rataj, Przekoracka‐Krawczyk, & Lubbe, 2017). This relates to “conceptual expansion,” one of the core cognitive operations that are at play in creativity (Rutter, Kröger, Hill, et al., 2012). Conceptual expansion describes our ability to stretch out the existing conceptual space to include new and possibly more remotely associated features (Ward, 1994, 2007). Fusing together remotely associated concepts or ideas is critical for creativity, but it is often cognitively costly. In this article we explore whether the cognitive effort associated with the comprehension of novel metaphors may be to some extent offset by prior domain knowledge. To get insight into the time course associated with cognitive processes underlying the novel metaphor comprehension we collected electrophysiological (EEG) data.

Studies investigating the electrophysiology of figurative language and metaphor comprehension have primarily focused on two Event‐Related Potentials (ERPs) components of EEG: (a) the N400, a negative wave with amplitude peak at around 400 ms that is known to reflect cognitive effort required to access meaning (Kutas & Federmeier, 2011; Kutas & Hillyard, 1980), and (b) later positive waves, the Late Positive Complex (LPC) or the semantic P600, known to index semantic re‐analysis and sentence‐level integration (Bornkessel‐Schlesewsky & Schlesewsky, 2008; Brouwer, Fitz, & Hoeks, 2012). In this paper, we focus on the N400 wave, because it has been shown to be a reliable index of conceptual expansion (Rutter, Kröger, Hill, et al., 2012) and figurative language comprehension (e.g., Arzouan, Goldstein, & Faust, 2007; Filik, Leuthold, Wallington, & Page, 2014; Goldstein, Arzouan, & Faust, 2012; Kazmerski, Blasko, & Dessalegn, 2003; Lai, Curran, & Menn, 2009; Rataj et al., 2017; Schneider et al., 2014). Due to inconsistencies in findings reporting figurative language comprehension in later processing stages (e.g., Arzouan et al., 2007; Filik et al., 2014; Rataj et al., 2017), the LPC/P600 analyses will be treated as exploratory.

The accumulating N400 evidence suggests that metaphors are more effortful to comprehend than literal language, and often less effortful than nonsensical language (e.g., Arzouan et al., 2007; Goldstein et al., 2012; Jankowiak, Rataj, & Naskręcki, 2017; Kazmerski et al., 2003; Lai et al., 2009; Rataj et al., 2017; Rutter, Kröger, Hill, et al., 2012; Schneider et al., 2014). For example, Arzouan et al. (2007) asked participants to perform a semantic judgment task on word pairs that consisted of either literal semantic relations (e.g., *burning fire*), conventional metaphorical relations (e.g., *lucid mind*), novel metaphorical relations (e.g., *ripe dream*), and nonsense semantic relations (e.g., *indirect blanket*). The results demonstrated a gradual modulation of the N400, with most pronounced N400 amplitudes to semantically unrelated word–pairs, followed by novel metaphorical, conventional metaphorical, and finally literal word–pairs. This graded N400 effect was interpreted in line with the *conceptual blending theory* (Coulson & Van Petten, 2002; Fauconnier & Turner, 1998), according to which metaphor comprehension requires construal of mappings between elements in remotely associated domains as well as the activation of background knowledge to enable their integration. Alternative theoretical accounts that explain how people arrive at a metaphorical meaning include the direct access view (e.g., Gibbs, 1994), graded salience view (e.g., Giora, 2003), and constraint‐satisfaction view (e.g., Katz, 2005; Katz & Ferretti, 2001; Pexman, 2008). An in‐depth discussion of these models is beyond the scope of the current manuscript. Instead, we focus on the *conceptual blending theory* as it has been particularly relevant in explaining the cognitive mechanisms underlying the creative power of metaphor.

Going beyond the domain of isolated word pairs, Rutter, Kröger, Hill, et al. (2012) used full sentences to tap into the electrophysiology of creative language comprehension. Participants were visually presented with novel metaphorical sentences (e.g., “The clouds have danced over the city”), literal sentences (e.g., “The clouds have moved over the city”), and nonsensical sentences (e.g., “The clouds have read over the city”); after reading they had to indicate whether each sentence was unusual (novel, original) and whether it was appropriate (sensical) in meaning, that is, the two fundamental features of creativity. For details on stimuli norming and selection, see Rutter, Kröger, Stark, et al. (2012). The behavioral results showed that participants took more time to answer to the “unusual” and “appropriate” question when reading novel metaphorical versus anomalous sentences and novel metaphorical versus literal sentences, respectively. The authors did not report the categorical data relating to the pattern of response to each question. Electrophysiological results showed increased N400 amplitudes to unusual and appropriate (novel metaphorical) sentences as well as unusual and inappropriate (anomalous) compared to usual and appropriate (literal) sentences. These findings replicated prior evidence demonstrating amplified N400 amplitudes to novel metaphorical sentences (Coulson & Van Petten, 2002, 2007; De Grauwe, Swain, Holcomb, Ditman, & Kuperberg, 2010; Jankowiak et al., 2017; Lai et al., 2009; Rataj et al., 2017; Schneider et al., 2014), lending further support for the conceptual blending theory. Critically, owing to a methodological approach inspired by creativity research, the study provided novel insight into the electrophysiology of sentence–level conceptual expansion, a crucial cognitive operation underlying creativity. Altogether, the available evidence suggests that comprehension of creative language, such as novel metaphors, is cognitively effortful as it requires building a coherent mental representation out of distant and remotely associated concepts.

Despite the available ERP evidence on novel metaphorical sentence processing, little is known about factors that may facilitate our understanding of novel metaphorical language and thus make us more efficient comprehenders of creative language. For example, Pynte, Besson, Robichon, and Poli (1996) demonstrated increased N400 amplitudes to unfamiliar metaphors that were preceded by irrelevant (Experiment 3) rather than relevant (Experiment 4) contexts. The authors also reported more reduced N400 amplitudes to unfamiliar metaphors preceded by a relevant context (Experiment 4) relative to familiar metaphors in the no‐context condition (Experiment 2). These results were interpreted in line with the *context‐dependent* account of metaphor processing suggesting that when metaphors are supported by relevant contexts, only their metaphorical meaning is accessed, short‐cutting the literal meaning (Pynte et al., 1996; also supportive of the direct access view, Gibbs, 1994). This would in turn reduce the cognitive effort associated with the co‐activation of literal and metaphorical meaning and thus result in the attenuation of the N400 amplitude. In a similar vein, Bambini et al. (2016) reported a reduction of the N400 for novel metaphorical sentences embedded in a supportive context (“That lawyer is really aggressive. He is a shark.”) relative to a minimal context (“Do you know what that lawyer is? A shark.”). Relatedly, Katz and Ferretti (2001) demonstrated that contextual information helped to disambiguate the processing of familiar and unfamiliar proverbs during online reading. Critically, these studies show that the cognitive effort required to comprehend figurative language may be mitigated when supported by relevant contextual information.

A potentially more potent contextual factor determining the efficiency of figurative language comprehension is one's personal knowledge. Specifically, given that prior knowledge has been found to profoundly impact language comprehension (as will be reviewed next), it is imperative to assess whether or not prior knowledge in a given domain facilitates comprehension of figurative language relating (or not) to that domain. Here, we address this question by collecting EEG data from engineering and nonengineering students reading novel metaphorical sentences that pertained to engineering concepts or to common concepts.

Prior knowledge has been argued to be the most important predictor of successful problem‐solving in a real‐life context (e.g., Ceci & Liker, 1986; Süß & Kretzschmar, 2018) and has been shown to greatly facilitate language comprehension (Chiesi, Spilich, & Voss, 1979; Ericsson & Kintsch, 1995; Ricks & Wiley, 2009; Spilich, Vesonder, Chiesi, & Voss, 1979; Voss, Vesonder, & Spilich, 1980; Wiley, George, & Rayner, 2018), irrespective of individuals' reading abilities (e.g., Recht & Leslie, 1988). For example, Katz and Pexman (1997) showed that knowledge about a speaker's sociocultural background (e.g., occupation) had an impact on interpreting a sentence as being metaphorical or ironic in meaning (see Katz, 2005 for a detailed discussion on the role of context in figurative language comprehension). Rodd et al. (2016) demonstrated that recent experience of rowing affected participants' language use, such that they activated rowing–related meanings of ambiguous words (e.g., “feather” or “square”) more often relative to participants with less or no recent rowing experience; this effect was amplified as a function of greater rowing experience. This finding shows that prior knowledge can boost an individual's ability to integrate new incoming information into the already existing representations more effectively, and thus can have a direct effect on language use. Moreover, having expert knowledge in a given domain may create too strong a bias, particularly in situations where problem‐solving requires going beyond one's field of expertise or when language comprehension relies on the activation of a subordinate, knowledge‐unrelated, meaning. Indeed, Wiley et al. (2018) found that baseball fans with extensive knowledge about the game found it more difficult to suppress the baseball–related dominant meaning of an ambiguous word (e.g., “bat,” referring to a baseball bat), even if the word appeared in a sentence context that favored the subordinate, baseball–unrelated, meaning (“bat,” referring to a flying mammal). This effect was not found among baseball fans who knew less about the game. This observation is highly relevant for creativity, whereby having expert knowledge in a domain may be at times constraining one's creative performance. Indeed, in a series of three experiments by Wiley (1998), participants with low knowledge of baseball outperformed baseball experts on a Remote Associates Test (RAT; Mednick, 1962) even when the experts were explicitly instructed to ignore any associations with baseball that a given item could create. Hence, although prior knowledge is believed to generally facilitate performance, these findings indicate that expert knowledge in a domain may sometimes constrain creative problem solving by narrowing and fixating the search for a solution too much toward a single domain of expertise.

In light of the abovementioned evidence, we set out to investigate if prior knowledge of engineering could facilitate comprehension of novel metaphorical sentences that related to engineering or to general knowledge. To this end, we invited 1st ‐year engineering and nonengineering students to take part in our experiment and asked them to make judgments about different types of sentences in terms of their originality and appropriateness (following Rutter, Kröger, Hill, et al., 2012) while they underwent EEG recording. Our engineering participants had a good foundation of engineering and majored in different fields of engineering, but, importantly, they were not engineering experts. To our knowledge, this is the first EEG study tapping into the possible impact of prior knowledge on creative language comprehension.

Following previous studies in the field of novel metaphor processing (e.g., Arzouan et al., 2007; Goldstein et al., 2012; Lai et al., 2009; Rataj et al., 2017; Tang, Qi, Jia, Wang, & Ren, 2017), our analyses focused on the N400 component of ERPs. Building on prior evidence, we expected an overall graded modulation of the N400 amplitudes, with most increased N400 to anomalous sentences, followed by novel metaphorical sentences, and literal sentences. Critically, if creative language use is affected by prior domain knowledge, it can be expected that engineers would show more attenuated N400 amplitudes to novel metaphors related to engineering than non‐engineers (i.e., an effect of prior knowledge). In contrast, processing of metaphors related to general knowledge is predicted to be comparable across both engineering and nonengineering groups. The analysis of later processing stages corresponding to the LPC/P600 time window will be exploratory.

## 2 METHOD

### 2.1 Participants

Forty‐three native English undergraduate students from a large American university gave informed consent to partake in the experiment that was approved by the university's IRB committee. Data from eight participants were discarded due to technical problems with some electrodes (*n* = 4), excessive alpha contamination leading to insufficient number of epochs per condition (*n* = 3), or having a first language other than English (*n* = 1).

The final sample included 35 undergraduates (*M*_age_ = 19.22; min = 18, max = 23): Eighteen engineering majors (*M*_age_ = 18.9; min = 18, max = 21; 10 females, 8 males) who were classified into the engineering group and 17 nonengineering majors (*M*_age_ = 19.5; min = 18, max = 23; 10 females, 7 males) who were classified into the nonengineering group. Engineering participants were enrolled in “Engineering 101” and declared their engineering majors prior to taking part in the experiment. All participants reported being right‐handed (Oldfield, 1971), having (corrected to) normal vision, and no history of neurological impairments.

### 2.2 Stimuli

Seventy‐six triplets of English sentences were constructed: 76 novel metaphorical sentences, 76 literal sentences, and 76 nonsensical sentences. Except for the main verb, sentences within each triplet were identical and thus also had the same sentence–final target word. Half the sentences in each of the three conditions related to engineering knowledge (*n* = 38) and half the sentences related to general knowledge (*n* = 38). The latter sentences were based on Rutter, Kröger, Hill, et al. (2012) and translated from German to English by a highly proficient German‐English bilingual. These sentences served as a control condition for the engineering sentences, but also to conceptually replicate and compare the present results with those of Rutter, Kröger, Hill, et al. (2012). All stimuli are available at Open Science Framework (OSF), https://osf.io/kx7bs/; example sentences are presented in Table 1.

**TABLE 1:** Examples of experimental material

Knowledge	Novel metaphorical	Literal	Anomalous
Engineering	The wind *tickled* the turbine	The wind *moved* the turbine	The wind *ate* the turbine
General	The earthquake *inhaled* the city	The earthquake *devastated* the city	The earthquake *defrosted* the city

The 228 critical verbs were matched for length and frequency of occurrence in American English (SUBTLEXus; Brysbaert, New, & Keuleers, 2012). This word frequency database contains part of speech information and includes verb‐specific frequency measures, which is particularly important for words that function as multiple grammatical categories (e.g., as verb and noun, such as “sleep”). A 3 (sentence type: metaphorical, literal, anomalous) × 2 (knowledge: engineering, general) by‐item ANOVA on word frequency values confirmed there was no effect of sentence type, *F*(2,222) = 2.02, *p* = .14, $\eta_{g}^{2}$ = 0.02, or knowledge, *F*(1,222) = 0.00, *p* = .98, $\eta_{g}^{2}$ < 0.0001, nor a sentence type × knowledge interaction, *F*(2,222) = 0.17, *p* = .84, $\eta_{g}^{2}$ = 0.002.

Prior to the experiment we conducted two sentence rating studies to establish the novelty (unusualness) and appropriateness (sensicality) of the novel metaphorical and literal sentences. Both rating studies were programed in E‐prime 2.0 (Psychology Software Tools, Inc.), which enabled us to track rating time and thus assess participants' engagement in the task. Rating times shorter than 2,000 ms were eliminated from further analyses (1.6% and 0.5% of all trials in novelty and appropriateness rating study, respectively). Overall, 6 out of 39 participants in the novelty rating study were excluded from further analyses due to too fast responses, incomplete responses, or adopting one pattern of responses for all trials.

The final participant sample for the novelty rating study included 33 native English speakers (*M*_age_ = 18.8; *SD*_age_ = 0.89; 15 females, 18 males; 11 engineering majors, 22 nonengineering majors). Participants were asked to rate novel metaphorical (*n* = 38) and literal (*n* = 38) sentences that related to engineering knowledge on a 4‐point scale for novelty/unusualness (1 highly unusual to 4 highly usual) and for appropriateness (1 highly inappropriate to 4 highly appropriate). Using the same procedure, other newly recruited 26 participants from the same population (*M*_age_ = 18.9; *SD*_age_ = 0.98; 20 females, 6 males) rated novel metaphorical (*n* = 38) and literal (*n* = 38) sentences relating to general knowledge (adopted from Rutter, Kröger, Hill, et al., 2012). In both rating studies, sentences were rotated across participants so that each participant rated only one meaning of each sentence frame.

Novelty and appropriateness ratings were each subjected to a 2 (sentence type: metaphorical; literal) × 2 (knowledge: engineering; general) ANOVA. The novelty analysis revealed a main effect of sentence type, *F*(1,150) = 200.3, *p* < .001, $\eta_{g}^{2}$ = 0.57, whereby novel metaphorical sentences (*M* = 2.21, 95% CI [2.10 2.32]) were rated as being more unusual than literal sentences (*M* = 3.32, 95% CI [3.21, 3.43]). Also, there was a main effect of knowledge, *F*(1,150) = 4.06, *p* = .046, $\eta_{g}^{2}$ = 0.03, with engineering items (*M* = 2.69, 95% CI [2.58 2.80]) being rated as more unusual than general knowledge items (*M* = 2.84, 95% CI [2.73 2.95]). The interaction did not differ from chance, *F*(1,150) = 3.18, *p* = .08, $\eta_{g}^{2}$ = 0.02.

**FIGURE 1:**
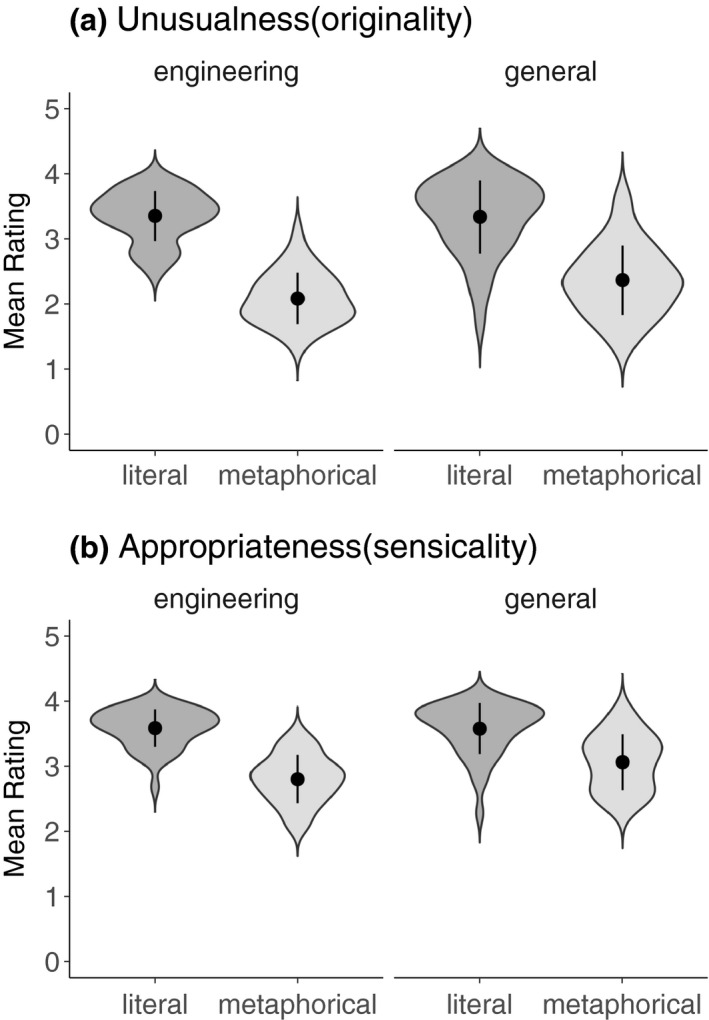
Mean unusualness (a) and appropriateness (b) ratings for literal and novel metaphorical sentences referring to engineering and general knowledge. Unusualness was rated on a 4‐point Likert scale, where 1 = highly unusual and 4 = highly usual. Appropriateness was rated on a 4‐point Likert scale, where 1 = highly inappropriate and 4 = highly appropriate

The appropriateness analysis showed a main effect of sentence type, *F*(1,150) = 114.8, *p* < .001, $\eta_{g}^{2}$ = 0.43, whereby novel metaphorical sentences were rated as being less appropriate (*M* = 2.92; 95% CI [2.84, 3.01]) than literal sentences (*M* = 3.57; 95% CI [3.49, 3.65]). Also, a main effect of knowledge was found, *F*(1,150) = 5.21, *p* = .024, $\eta_{g}^{2}$ = 0.03, with engineering items (*M* = 3.18, 95% CI [3.09, 3.26) being rated as less appropriate than general knowledge items (*M* = 3.32, 95% CI [3.23, 3.40). Finally, the sentence type × knowledge interaction, *F*(1,150) = 4.88, *p* = .029, $\eta_{g}^{2}$ = 0.03, revealed that novel metaphorical sentences referring to engineering knowledge were rated as being less appropriate (*M* = 2.79; 95% CI [2.67, 2.91]) than those referring to general knowledge (*M* = 3.06; 95% CI [2.94, 3.18], at *p* = .003). There was no difference between literal sentences referring to engineering (*M* = 3.57, 95% CI [3.45, 3.69]) and general knowledge (*M* = 3.58, 95% CI [3.46, 3.70]; *p* > .05). Figure 1 depicts a graphical representation of the unusualness (1a) and appropriateness (1b) rating data.

### 2.3 Procedure

Participants were seated approximately 100 cm away from the screen in a dimly lit and sound–attenuated booth. During EEG cap preparation, participants completed a Language History Questionnaire and Edinburgh Handedness Questionnaire (Oldfield, 1971). Participants were instructed that in each trial they would be reading a sentence presented word by word at the center of the screen and that their task was to decide upon seeing the last word whether or not the sentence was unusual (original) in meaning followed by a question whether or not it was appropriate (sensical) in meaning, by pressing one of two buttons. Participants were instructed to respond “yes” to the “Unusual?” question if they thought the sentence sounded novel or unfamiliar to them, and “no” if it sounded familiar or known. They were instructed to say “yes” to the “Appropriate” question when they thought the sentence sounded sensible and sensical to them, and “no” if it sounded nonsensical.

Each trial began with the presentation of a fixation signal (500 ms), after which words were displayed one by one at the center of the screen at a rate of 300 ms with an ISI of 250 ms. After each sentence, two questions, “Unusual?” and “Appropriate?”, were displayed sequentially and remained on the screen until the participant's response. After each trial, participants could take a break and proceed to the next trial by pressing a designated button. Participants completed a practice session and three blocks of experimental trials. Sequence of trials was randomized within each block; block order and response keys were counterbalanced across participants.

### 2.4 EEG acquisition and analysis

Electrophysiological data were continuously recorded in reference to electrode FCZ at a rate of 500 Hz from 30 Ag/AgCl active ActiCAP electrodes (Brain Products GmbH, Germany) placed according to the extended 10–20 convention (Jasper, 1958: Fp1, Fp2, F7, F3, Fz, F4, F8, FC5, FC1, FC2, FC6, T7, C3, Cz, C4, T8, CP5, CP1, CP2, CP6, P7, P3, Pz, P4, P8, PO9, O1, Oz, O2, PO10). Additionally, an electrode was placed on each mastoid. The vertical and horizontal electro‐oculograms (EOGs) were recorded from electrodes located above and below the left eye and at the outer canthus of each eye. Impedances were kept below 10 kOhms at all electrode sites. EEG signals were amplified with a Neuroscan SynAmps2 amplifier unit (El Paso, TX) and filtered online with a band pass filter between 0.05 and 200 Hz.

All pre‐processing steps and analyses were performed using EEGLAB (v14.1.1; Delorme & Makeig, 2004) and ERPLAB (v6.1.4; Lopez‐Calderon & Luck, 2014) toolboxes in Matlab R2017a (The MathWorks, Inc.). Continuous EEG data was band‐pass filtered between 0.1 and 30 Hz using a 2nd order noncausal IIR Butterworth digital filter (12 dB/octave roll‐off). Unsystematic artifacts in continuous EEG data caused by muscle activity were detected and removed during manual inspection. Bad channels were identified via visual inspection and with the help of a TrimOutlier plugin (Lee & Miyakoshi; https://sccn.ucsd.edu/wiki/TrimOutlier), by excluding channels with a standard deviation >100 μV and <1 μV (*M = *1.8, min = 1, max = 4). Continuous data were re‐referenced to the algebraic mean of activity over the left (M1) and right (M2) mastoids.

For the purpose of the Independent Component Analysis (ICA) only, a copy of the original data was high‐pass filtered at 1Hz (cut‐off frequency, 0.5Hz) using Hamming windowed sinc FIR filter (filter order: 1650), and CleanLine plugin (Mullen, 2012) was used to reduce line noise at 60Hz. Applying a high‐pass filter to EEG signal is recommended for ICA as it prevents low frequency drifts from dominating the ICA decomposition (Beese, Meyer, Vassileiou, & Friederici, 2017; Winkler, Debener, Müller, & Tangermann, 2015; Wu et al., 2018). Extended infomax ICA (Lee, Girolami, & Sejnowski, 1999; as implemented in EEGLAB) was performed on the high‐pass filtered continuous EEG, and the obtained ICA weights were then copied to the original 0.1–30 Hz band‐pass filtered data (for a similar procedure, see Baldwin et al., 2017; Rabbitt, Roberts, McDonald, & Peterson, 2017; Schirmer & Gunter, 2017). ICs containing ocular and muscle artifacts as well as electrode pops were removed from the data (*M* = 3.2; min = 2, max = 5). Following ICA, missing channels were interpolated using the spherical spline method implemented in EEGLAB.

The data were subsequently segmented into 1,000 ms final word (noun) epochs (−200 to 800 ms) and 800 ms medial word (verb) epochs (−200 to 600 ms).^1^For graphical purposes, we selected a slightly bigger epoch window for verb analysis (600 ms instead of 550 ms). This choice did not affect the pattern of EEG responses. The Mass Univariate Analysis showed that the N400 to verbs was constrained to the 350 – 500 ms time window. Baseline period was corrected relative to prestimulus activity. All epochs with activity exceeding ±100 μV at any electrode site were automatically rejected using a peak‐to‐peak moving window (window size: 200 ms; window step: 100 ms) procedure in ERPLAB (rejected noun epochs: *M*_engineers_ = 1.4, min = 0, max = 6; *M*_nonengineers_ = 1.3, min = 0, max = 6; rejected verb epochs: *M*_engineers_ = 0.9, min = 0, max = 8; *M*_nonengineers_ = 0.8, min = 0, max = 6). Finally, averaged ERP waveforms for nouns and verbs were computed from the epoched EEG data.

**FIGURE 2:**
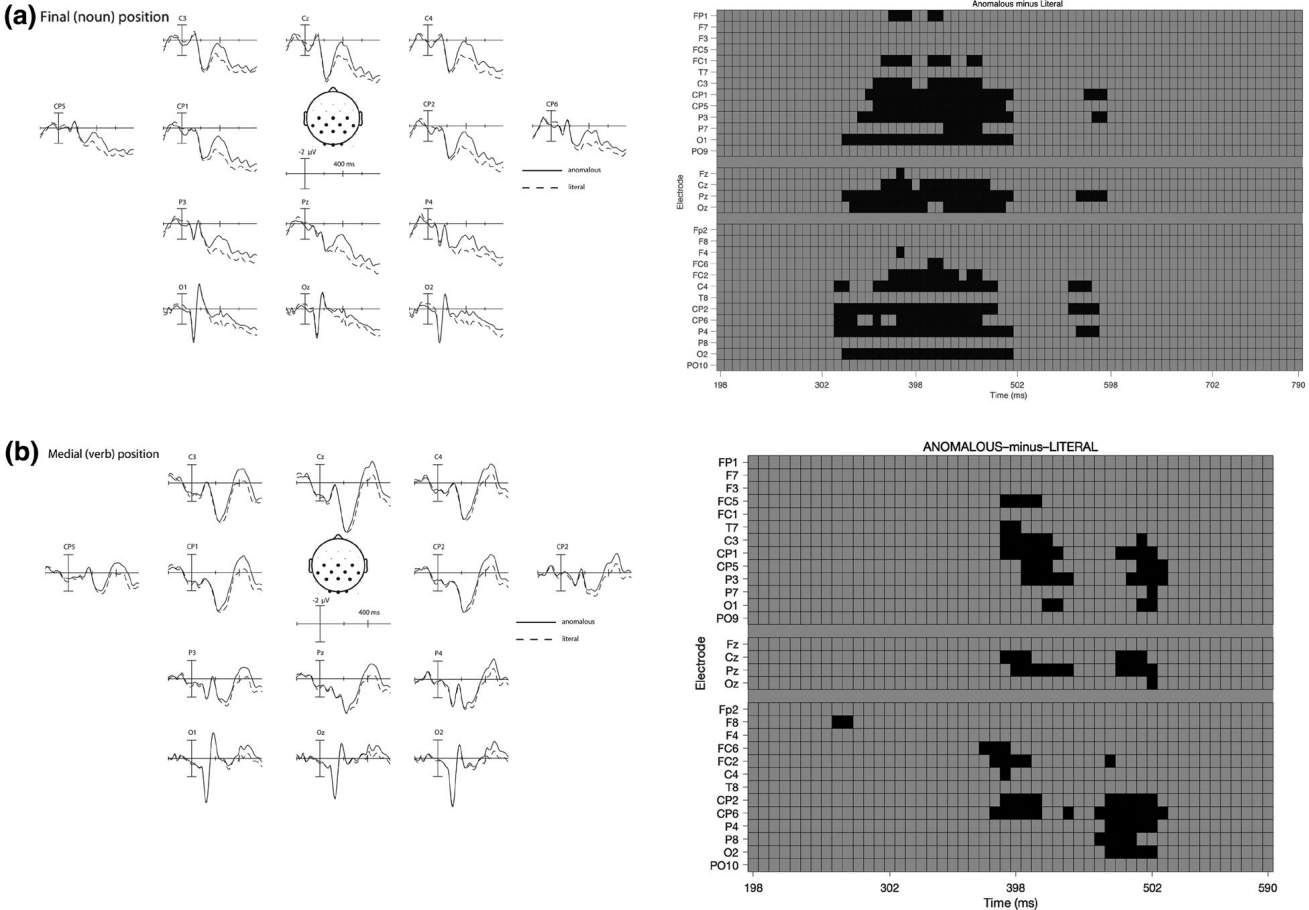
Averaged ERP waves (left panel) and raster plots (right panel) for nouns (a) and verbs (b) displaying the results of a *t*‐max permutation test in a two‐dimensional grid from mass univariate analyses of the anomalous minus literal comparison of the ERP data collapsed across the two groups. Significant *t*‐tests for negative ERP differences are represented in black. No positive ERP differences were observed

We focused on two ERP components: the N400 and the semantic P600/LPC. To establish a common topography and timing of the N400 and semantic P600/LPC for both engineering and nonengineering participants, we ran *t*‐max permutation tests in Mass Univariate Analysis toolbox (Groppe, Urbach, & Kutas, 2011) on the literal–anomalous difference wave, where we predicted a maximal effect (cf., Luck & Gaspelin, 2017). The ERPs from the critical condition were submitted to a repeated‐measures, two‐tailed permutation test based on the tmax statistic (described in Blair & Karniski, 1993) using a family wise alpha level of 0.05, to detect reliable mean difference of amplitudes in the time windows between 200 and 800 ms (noun) and 200 and 600 ms (verb) poststimulus onset. This procedure is characterized by a strong control of familywise error rate, providing the same degree of false discovery rate as a Bonferroni correction (see Groppe et al., 2011, for more detailed discussion). In each case, the null distribution was derived from 2,500 within‐subject random permutations. As a result of this procedure the N400 was analyzed at 13 centro‐parietal electrodes (C3, Cz, C4, CP5, CP1, CP2, CP6, P3, Pz, P4, O1, Oz, O2) where the effect was found to be maximal for both nouns and verbs (see Figure 2). However, the timing of the N400 for word‐medial and word‐final positions was found to be slightly different, with maximal effects found between 300 and 500 ms for word‐final and 350–500 ms for word‐medial positions. Finally, the Mass Univariate Analysis did not reveal any differences in a later time window (including the semiantic P600/LPC time window), and hence from now on we focus solely on the N400 effect.

Further statistical analyses were conducted by means of 3 (sentence type: metaphorical, anomalous, literal) × 2 (knowledge: engineering, general) × 2 (group: engineers, non‐engineers) repeated measures ANOVAs, with the mean amplitude of N400 as a dependent variable, sentence type, and knowledge as within‐subject independent variables, and group as a between‐subject variable. A Greenhouse–Geisser correction was applied where applicable and *p* values obtained from post hoc pairwise comparisons were adjusted using the Bonferroni correction. For effect sizes, we report generalized eta‐squared that has been argued to be preferred over partial eta‐squared, particularly when dealing with between‐subject designs (see, Lakens, 2013; Olejnik & Algina, 2003). Following Cohen's (1988) benchmarks, small $\eta_{g}^{2}$ = 0.01; medium $\eta_{g}^{2}$ = 0.06; large $\eta_{g}^{2}$ = 0.14.

### 2.5 Behavioral analysis

Estimates are based on logit linear mixed models (Jaeger, 2008) using the lme4 package (Version 1.1–23; Bates, Maechler, Bolker, & Walker, 2015) in the R environment (Version 4.0; R Core Team, 2020). We created two separate models to analyze the categorical responses to the “unusual” and “appropriate” question. We included the following fixed‐effects in each model, (a) sentence type (literal, anomalous, metaphorical), (b) knowledge (engineering knowledge, general knowledge), (c) group (engineers, non‐engineers), and their interactions. All fixed effects were coded using contrast coding; as such sentence type was included as two separate fixed effects in a predictive manner (for a discussion, see Schad, Vasishth, Hohenstein, & Kliegl, 2020). For the “unusual” question we included the contrast between metaphorical and anomalous sentences (*metaphorical‐anomalous*), as well as literal versus metaphorical and anomalous (*literal‐metaphorical_anomalous*) sentences. This enabled us to test two predictions: (a) anomalous relative to metaphorical sentences should be perceived as more unusual (more “yes” responses to the “unusual” question); (b) anomalous and metaphorical sentences relative to literal should be perceived as more unusual. For the “appropriate” question we tested the same contrasts: (a) metaphorical versus anomalous (*metaphorical‐anomalous*) as well as literal versus metaphorical and anomalous (*literal* – *metaphorical_anomalous*) sentences. These contrasts tested the following predictions: (a) metaphorical sentences would be perceived as more appropriate (sensical) than anomalous sentences (more “yes” responses to the “appropriate” question); (b) literal sentences would be perceived as more appropriate than metaphorical and anomalous sentences.

For both models, we first computed generalized linear mixed models (GLMM) with a full random‐effect structure, including subject‐ and item‐related variance components for intercepts and maximal by‐subject and by‐item random‐slopes for fixed‐effects (Barr, Levy, Scheepers, & Tily, 2013). Both maximal models turned out to be too complex and not supported by the data. Therefore, we selected parsimonious GLMMs following the recommendations by Bates et al. (2015; see also Matuschek, Kliegl, Vasishth, Baayen, & Bates, 2017). Small variance parameters were removed using the lme4::rePCA and lme4::VarCorr functions until both GLMMs were supported by the data. This resulted in two separate models for responses to the “unusual” and “appropriate” questions:

1. Unusualness ~ Sentence*Knowledge*Group + (1 + Sentence |Subject) + (1 + Group|Item).
2. Appropriateness ~ Sentence*Knowledge*Group + (1 + Knowledge|Subject) + (1|Item).

*β* estimates and significance of fixed effects and interactions (*p* values) are based on Laplace approximation (using the *lmerTest* package; Kuznetsova, Brockhoff, & Chritensen, 2017). Both model summaries are available at https://osf.io/kx7bs/.

## 3 RESULTS

### 3.1 Responses to “unusual?” question

**FIGURE 3:**
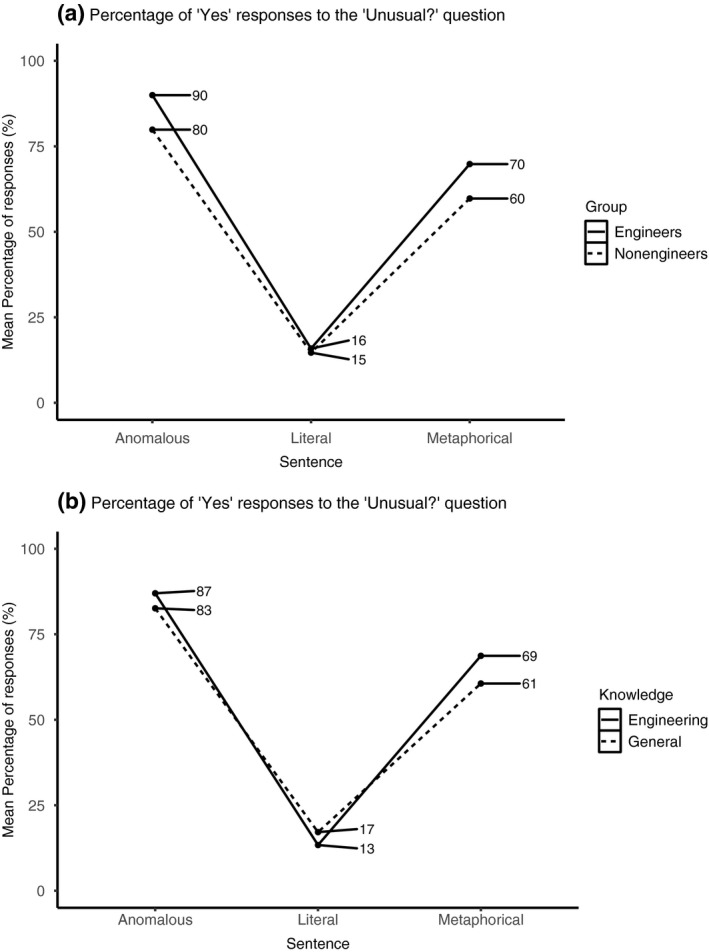
Mean percentage of “Yes” responses to the “Unusual?” question for each group (a) and for each sentence type (b)

A fixed effect of sentence type showed that anomalous sentences were perceived as more unusual than metaphorical sentences (*β* = 1.60, *SE* = 0.22, *z* = 7.29, *p* < .001). Also, metaphorical and anomalous sentences were perceived as more unusual than literal sentences (*β* = 5.09, *SE* = 0.30, *z* = 16.88, *p* < .001). A fixed effect of group showed that engineers perceived sentences as more unusual (*β* = 0.59, *SE* = 0.22, *z* = −2.65, *p* = .008) than non‐engineers. A sentence type × group interaction (Figure 3a) showed that engineers relative to non‐engineers perceived anomalous and metaphorical sentences as more unusual than literal sentences (*β* = 1.21, *SE* = 0.45, *z* = 2.71, *p* = .007). A sentence type × knowledge (Figure 3b) interaction showed that metaphorical and anomalous sentences were perceived as more unusual than literal sentences when both types of sentences referred to engineering rather than general knowledge (*β* = 1.08, *SE* = 0.44, *z* = 2.44, *p* = .015). No other effects were found.

### 3.2 Responses to “appropriate?” question

**FIGURE 4:**
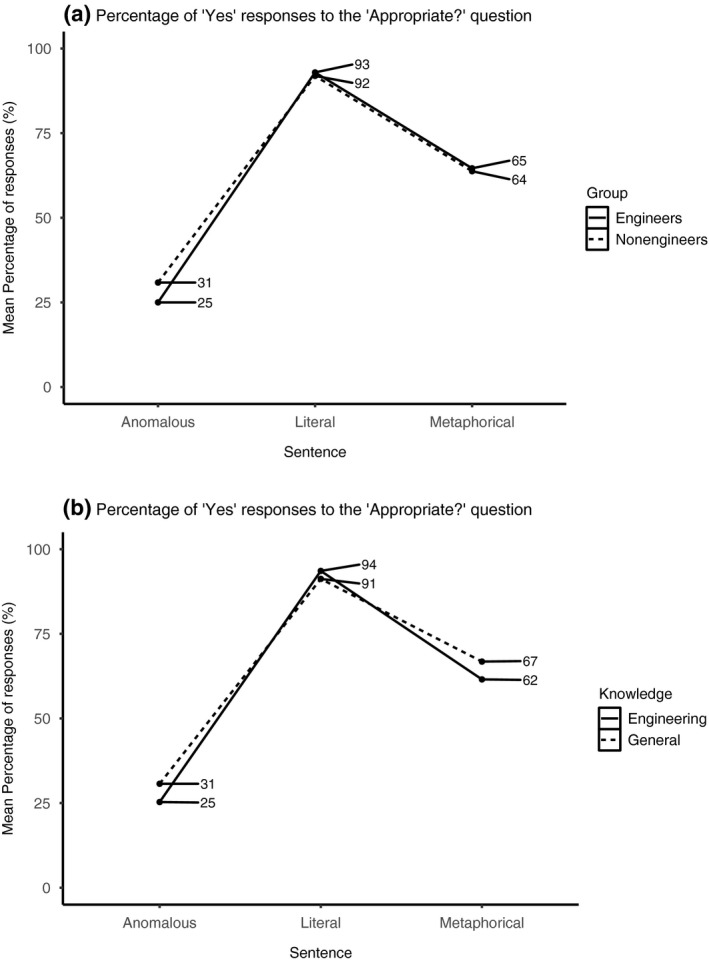
Mean percentage of “Yes” responses to the “Appropriate?” question for each group (a) and for each sentence type (b)

A fixed effect of sentence type showed that metaphorical sentences were perceived as more appropriate than anomalous sentences (*β* = 2.01, *SE* = 0.17, *z* = 11.75, *p* < .001). Also, literal sentences were perceived as more appropriate than metaphorical and anomalous sentences (*β* = 4.55, *SE* = 0.22, *z* = 20.56, *p* < .001). A sentence type × group interaction (Figure 4a) demonstrated that non‐engineers relative to engineers perceived anomalous sentences as more appropriate (*β* = 0.48, *SE* = 0.13, *z* = 3.74, *p* < .001); also, the difference in “yes” responses to the appropriate question for literal sentences as compared to metaphorical and anomalous sentences was more pronounced in engineers (*β* = −0.55, *SE* = 0.23, *z* = −2.41, *p* < .016). Finally, a sentence type × knowledge (Figure 4b) interaction showed that literal sentences were perceived as more appropriate than metaphorical and anomalous sentences, particularly when both sentence types referred to general knowledge (*β* = 1.03, *SE* = 0.44, *z* = 2.34, *p* < .020). No other effects were found.

### 3.3 N400 (mid–sentence verb)

**FIGURE 5:**
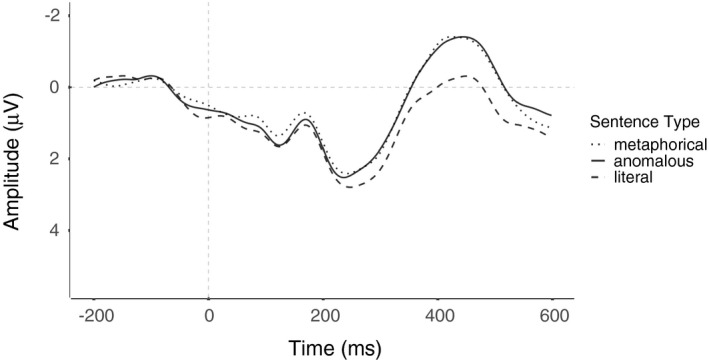
ERPs elicited by verbs embedded in novel metaphorical, anomalous, and literal sentences collapsed across both engineers and non‐engineers. All waveforms represent brain potential variations computed via linear derivation from 13 centro‐parietal electrodes (C3, Cz, C4, CP5, CP1, CP2, CP6, P3, Pz, P4, O1, Oz, O2)

The ANOVA showed a main effect of sentence type, *F*(1.82, 60.09) = 14.36, *p* < .001, $\eta_{g}^{2}$ = 0.1, whereby the mid–sentence verbs elicited a more negative N400 amplitude for both the anomalous (*M* = −1.08, 95% CI [−1.4 −0.68]) and novel metaphorical (*M* = −1.08, 95% CI [−1.4 −0.68]) relative to literal sentences (*M* = −0.05, 95% CI [−0.45 0.34]; *t*_met‐lit_(66) = −4.64, *SE* = 0.22, *p* < .001; *t*_ano‐lit_(66) = −4.65, *SE* = 0.22, *p* < .001), with no differences found for anomalous and novel metaphorical sentences (*t*(66) = 0.01, *SE* = 0.22, *p* = 1). For a visual presentation of this effect, see Figure 5. Also, the main effect of group was marginally significant, *F*(1,33) = 3.7, *p* = .063, $\eta_{g}^{2}$ = 0.04, with marginally more negative N400 amplitudes in the engineering (*M* = −1.04, 95% CI [−1.5, −0.6]) than nonengineering (*M* = −0.45, 95% CI [−0.9, 0.0]) students. No other effects were found.

### 3.4 N400 (sentence–final noun)

**FIGURE 6:**
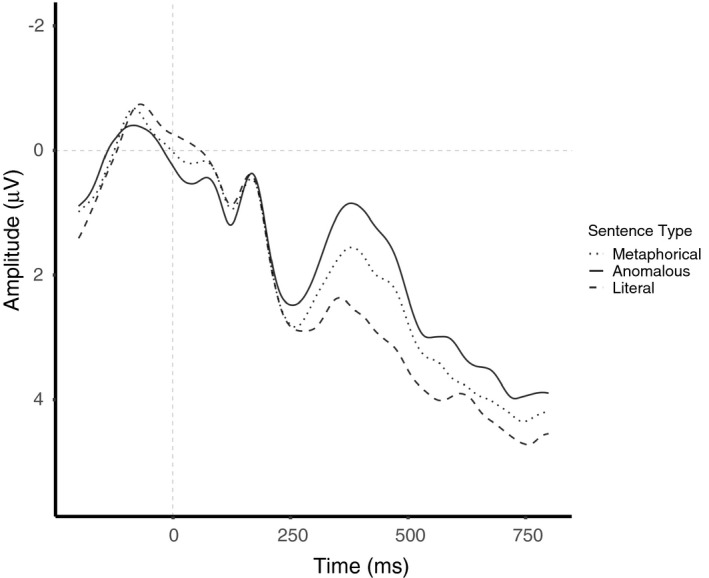
ERPs elicited by sentence‐final words embedded in novel metaphorical, anomalous, and literal sentences collapsed across both engineers and non‐engineers. All waveforms represent brain potential variations computed via linear derivation from 13 centro‐parietal electrodes (C3, Cz, C4, CP5, CP1, CP2, CP6, P3, Pz, P4, O1, Oz, O2)

The ANOVA showed a main effect of sentence type, *F*(1.87, 61.78) = 19.81, *p* < .001, $\eta_{g}^{2}$ = 0.05, showing a gradual decrease in the N400 amplitude as a factor of sentence type: Anomalous sentences elicited the most negative N400 amplitude (*M* = 1.30, 95% CI [.44 2.15]) that differed from both novel metaphorical sentences (*M* = 1.93, 95% CI [1.07 2.78]; *t*(66) = 2.67, *SE* = 0.24, *p* = .029), and literal sentences (*M* = 2.78, 95% CI [1.93 3.63]; *t*(66) = −6.27, *SE* = 0.24, *p* < .001). Also, the N400 amplitude to novel metaphorical sentences was more negative than that of literal sentences (*t*(66) = −3.60, *SE* = 0.24, *p* = .002). For a visual presentation of this effect, see Figure 6.

**FIGURE 7:**
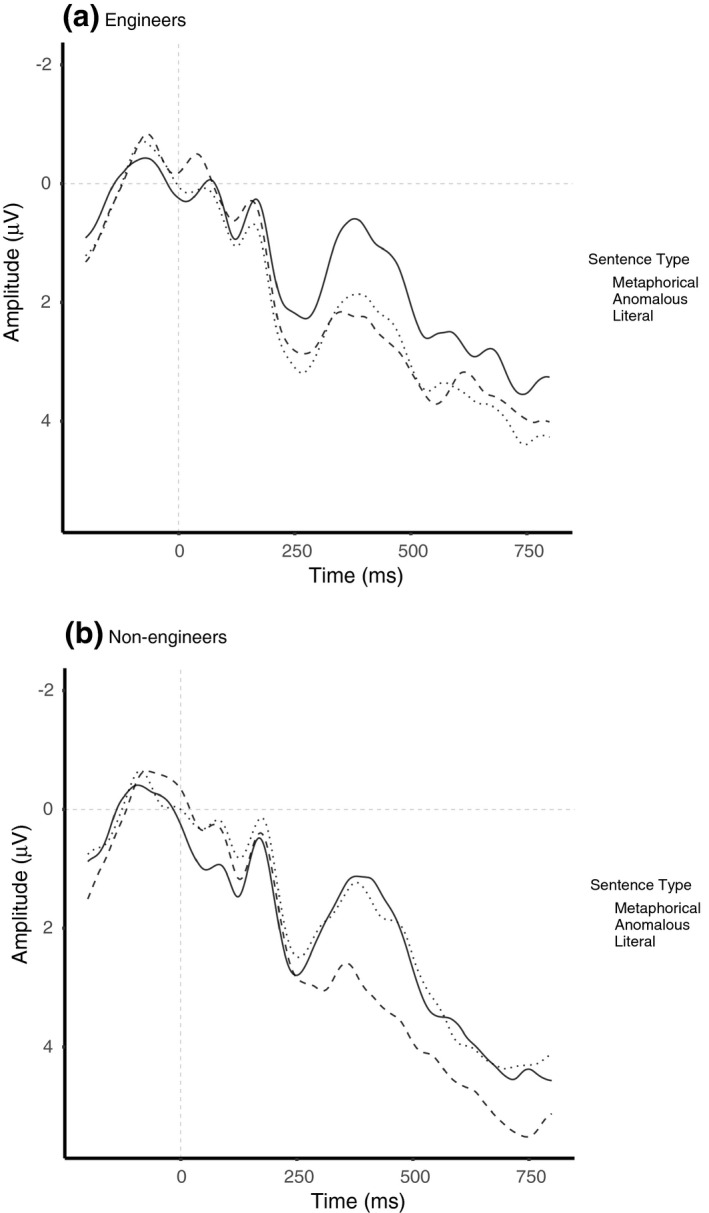
ERPs elicited by sentence‐final words embedded in novel metaphorical, anomalous, and literal sentences in engineers (a) and non‐engineers (b). All waveforms represent brain potential variations computed via linear derivation from 13 centro‐parietal electrodes (C3, Cz, C4, CP5, CP1, CP2, CP6, P3, Pz, P4, O1, Oz, O2)

The analysis further revealed asentence type × group interaction, *F*(1.87,61.78) = 3.71, *p* = .033, $\eta_{g}^{2}$ = 0.011, with differences in N400 amplitude in engineering students between anomalous (*M* = 1.06, 95% CI [−0.14 2.26]) and novel metaphorical (*M* = 2.19, 95% CI [.99 3.40]; *t*(66) = 3.43, *SE* = 0.33, *p* = .003) as well as anomalous and literal (*M* = 2.44, 95% CI [1.24 3.64]; *t*(66) = −4.18, *SE* = 0.33, *p* < .001) sentences, with no differences in N400 amplitude between metaphorical and literal sentences (*t*(66) = −0.76, *SE* = 0.33, *p* = 1). In contrast, N400 amplitudes in nonengineering students differed between anomalous (*M* = 1.53, 95% CI [.32 2.73]) and literal (*M* = 3.12, 95% CI [1.91 4.33]; *t*(66) = −4.68, *SE* = 0.34, *p* < .001), as well as novel metaphorical (*M* = 1.66, 95% CI [.45 2.87]) and literal sentences (*t*(66) = −4.29, *SE* = 0.34, *p* < .001), with no such differences between anomalous and novel metaphorical sentences (*t*(66) = 0.39, *SE* = 0.34, *p* = 1).^2^an additional analysis on the appropriate responses only yielded the same pattern of results. For a visual presentation of this interaction, see Figure 7.

**FIGURE 8:**
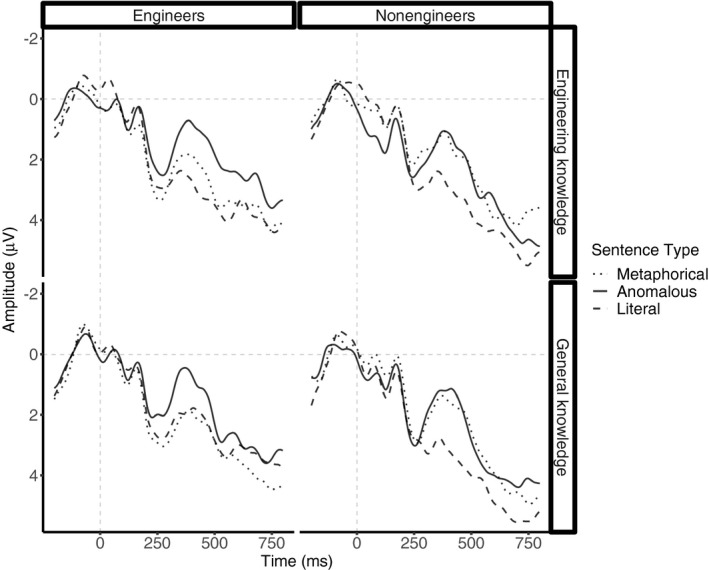
ERPs elicited by sentence‐final words embedded in novel metaphorical, anomalous, and literal sentences split by group (Engineers, Non‐engineers) and knowledge (Engineering knowledge, General knowledge). All waveforms represent brain potential variations computed via linear derivation from 13 centro‐parietal electrodes (C3, Cz, C4, CP5, CP1, CP2, CP6, P3, Pz, P4, O1, Oz, O2)

Although the analyses did not reveal a sentence type × knowledge ×group interaction, *F*(1.90, 62.81) = 1.09, *p* = .3, $\eta_{g}^{2}$ = 0.002, we inspected whether engineers differed from non‐engineers in how they processed novel metaphorical sentences relating to engineering, in line with our a priori hypothesis. A Welch independent two‐sample *t* test confirmed more reduced N400 amplitudes to novel metaphorical sentences relating to engineering concepts in engineers (*M* = 2.2, 95% CI [.90, 3.43]) relative to non‐engineers (*M* = 1.6, 95% CI [.33, 2.87]), *t*(384.6) = 2.06, *p* = .04. For a graphical representation of this pattern, see Figure 8.

## 4 DISCUSSION

This study set out to investigate if prior domain knowledge modulates EEG responses to novel metaphorical sentences that provide an index of linguistic creativity (cf., Beaty, Silvia, & Benedek, 2017; Rataj et al., 2017; Rutter, Kröger, Hill, et al., 2012). Based on prior findings suggesting that expert domain knowledge might help or hinder creative performance (e.g., Wiley, 1998; Wiley et al., 2018), we recruited 1st ‐year engineering students with a good foundation but not (yet) expert knowledge in engineering and a control group of nonengineering students for our experiment.

Our results demonstrate a graded modulation of the N400 component, with most pronounced N400 to anomalous sentences, followed by novel metaphorical sentences, and anomalous sentences. Critically, however, this effect seems to have been driven by a differential modulation of the N400 amplitude to novel metaphorical sentences in engineers and non‐engineers. Specifically, while engineering students demonstrated increased N400 to anomalous relative to novel metaphorical and literal sentences (with no observed N400 difference between metaphorical and literal sentences), non‐engineers showed a mirror effect for metaphorical sentences, with more pronounced N400 to anomalous and novel metaphorical sentences relative to literal sentences. Essentially, novel metaphorical sentences patterned with literal sentences in the case of engineers and with anomalous sentences in the case of non‐engineers. This finding suggests that when engineers were presented with novel metaphorical sentences, they seemed to construe mappings between remotely associated concepts more effectively and with less cognitive effort than non‐engineers. We propose that this effect may be driven by the fact that engineering students had at their disposal background domain knowledge of both engineering and common concepts that enabled activating relevant concepts in semantic memory and arriving at the interpretation of novel metaphors more efficiently. In contrast, for nonengineering students engineering metaphors were, at best, distantly related to their conceptual knowledge. This interpretation is thus consistent with the conceptual blending theory (Coulson & Van Petten, 2002; Fauconnier & Turner, 1998), according to which metaphor comprehension requires not only the construal of mappings between elements in remotely associated domains but also the activation of domain‐specific knowledge to enable integration between two distant domains.

It seems that the availability and activation of background knowledge may be particularly beneficial for the comprehension of novel metaphors whose understanding requires establishing a *new* relationship between two remotely associated domains. This may explain why studies to date have repeatedly found no N400 amplitude differences between novel metaphors and anomalous sentences (e.g., Arzouan et al., 2007; Goldstein et al., 2012; Lai et al., 2009; Rataj et al., 2017; Rutter, Kröger, Hill, et al., 2012), which is precisely what we have demonstrated here for nonengineering students. Establishing new connections between distant domains when comprehending a novel metaphor that relates to non‐existent, or at the very least less accessible, concepts in semantic memory seems to be comparable to the effort needed to make sense out of an anomalous sentence. This, however, may not be the case when the novel metaphor relates to specific domain knowledge that aligns with the comprehender's prior knowledge, as we suggest is the case in engineering students in our study. Indeed, a priori planned comparisons carried out to investigate N400 amplitude difference between novel metaphorical sentences relating to engineering knowledge in engineering and nonengineering students supported our prediction about the effect of prior knowledge, demonstrating less negative (i.e., attenuated) N400 amplitudes to engineering metaphors in the engineering than the nonengineering group. Note, however, that this effect happened in the absence of the expected three‐way interaction between group, sentence type, and knowledge, and thus should be interpreted with caution. Alternatively, it may also be the case that having at their disposal domain and general knowledge engineering students performed overall better than nonengineering students in the processing of both types of novel metaphors, with a possibility that the latter group was additionally confused by engineering sentences overall and thus performing worse on metaphorical language tapping into both engineering and general knowledge concepts.

Our findings also contribute to the idea that the N400 is expected to be attenuated for conceptual mappings that are more easily accessed (Chwilla, Brown, & Hagoort, 1995; Kutas & Federmeier, 2011). It seems that engineering students processed novel metaphorical sentences relating to general and engineering‐related knowledge with relative ease, because they were equipped with prior knowledge from both domains. This claim is further supported by behavioral data showing that engineers relative to non‐engineers more frequently identified novel metaphorical sentences as having an unusual, original meaning. It seems that having prior knowledge of engineering combined with general knowledge may have given our engineers an advantage in the processing of all types of sentences, which was not the case for nonengineering students.

When interpreted in the context of creativity research, the reported attenuated N400 amplitude to novel metaphors, specifically those relating to engineering knowledge, in engineering versus nonengineering students may reflect that having prior domain knowledge puts an individual at an advantage of being able to more efficiently establish novel semantic relationships in the process of conceptual expansion, and thus interpret figurative language with greater ease. It is, therefore, possible that prior domain knowledge facilitates the cognitive operation of conceptual expansion by making domain‐related conceptual information more readily accessible. According to Boden (2004), the diversity of an individual's conceptual space critically contributes to the richness of their mental resources for combining the available concepts into novel ideas. Equipped with general and engineering concepts, an individual may thus more readily combine concepts from those two domains to create something new (i.e., *combinational creativity* in Boden's terms). Essentially, prior knowledge may be seen as providing building blocks for the generation as well as interpretation of novel ideas.

Prior knowledge may also function as highly relevant contextual information, helping to resolve the ambiguity induced by the blending of remotely associated or completely unrelated concepts. This is in line with prior research demonstrating that contextual information can effectively mitigate the cognitive effort associated with comprehending novel metaphorical language by reducing the amplitude of the N400 (Bambini et al., 2016; Pynte et al., 1996). In a similar vein, Nieuwland and Van Berkum (2006) demonstrated that increased N400 amplitudes to animacy violation, as in “The girl comforted the clock,” disappeared when the sentence was embedded in a wider, supportive context of a fictional story about a clock that was feeling depressed. Altogether, prior knowledge may be seen as providing background contextual information that helps to resolve ambiguities in creative language comprehension.

Note that our behavioral data support the observed N400 effect. Engineers relative to non‐engineers turned out to identify the originality and novelty of presented sentences more frequently, particularly novel metaphorical and anomalous sentences. At the same time when judging the meaningfulness of sentences, engineers tended to more frequently show a response pattern that was in line with our predictions. This demonstrates that when reading novel metaphorical sentences engineering students may have better identified the unusualness (novelty, originality) and appropriateness (sensicality) of metaphors.

Finally, the mass univariate analysis did not show any differences between the conditions in the later sematic P600/LPC window and thus we decided against pursuing the analyses further. Some prior studies examining novel metaphor processing also did not find differences in this late positivity window (e.g., Arzouan et al., 2007; Lai et al., 2009; Pynte et al., 1996; Rutter, Kröger, Hill, et al., 2012; Tang et al., 2017). Generally, studies reported inconsistent findings with either increased (e.g., Bambini et al. 2016, De Grauwe et al., 2010) or decreased (e.g., Arzouan et al., 2007; Goldstein et al., 2012; Rataj et al., 2017) LPC to metaphors relative to literal and anomalous structures. These discrepancies have been argued to arise from the degree of conventionality of experimental materials, with novel metaphors leading to a decreased LPC (behaving more like anomalies) while conventional metaphors eliciting an increased LPC, relative to literal and anomalous sentences. Indeed, Goldstein et al. (2012) observed a decreased LPC for unexplained novel metaphors and an increased LPC for explained novel metaphors. This could account for the lack of an LPC modulation in the present study. In contrast, here we observe an extended or sustained negativity resulting from the N400 modulation that continues into the later time window without a significant change in the relative amplitude difference between critical conditions (elsewhere a similar effect was referred to as late negativity, e.g., Arzouan et al., 2007; Tang et al., 2017). A similar sustained negativity was reported by Rutter, Kröger, Hill, et al. (2012) in response to novel metaphorical and anomalous sentences, relative to literal sentences. The authors interpreted this effect as being reflective of a sustained effort to integrate the meaning of two remotely associated concepts that could not be resolved within the time window of semantic access (i.e., the N400 window). Hence, the N400 effect and the negativity that followed could be interpreted as a single sustained effect.

### 4.1 Conclusion

This study set out to explore if and how prior domain knowledge affects the processing of novel metaphorical language, an index of linguistic creativity. Engineering and nonengineering students read literal, anomalous, and novel metaphorical sentences that related to engineering or general knowledge. Engineers processed novel metaphorical sentences with greater ease than non‐engineers regardless of which knowledge concepts pertained to (i.e., engineering‐related or general knowledge sentences). We argued that, contrary to non‐engineers, engineers' novel metaphor comprehension could benefit from the activation of both engineering‐related and general knowledge concepts, which resulted in a significant advantage in their interpretation of novel metaphorical language. This study is the first to show a direct impact of prior knowledge on the interpretation of novel metaphorical language, and as such contributes to, and bridges, the fields of novel metaphor processing and creativity research. It remains to be tested if the effect reported in this study extends to contexts other than engineering, and whether prior knowledge also affects the production of new metaphors or other forms of creative language (cf., Beaty et al., 2017; Benedek et al., 2014).

## AUTHOR CONTRIBUTIONS

*Study concept and design*: Jończyk, van Hell, Kremer, and Siddique. *Data collection and analyses*: Jończyk. *Interpretation of data*: Jończyk, van Hell. *Drafting of the manuscript*: Jończyk. *Critical revision of the manuscript for important intellectual content*: van Hell, Kremer, and Siddique. *Statistical analysis*: Jończyk.

## Data Availability

The data collected and analyzed for the purpose of the current study is available from the corresponding author upon reasonable request. The stimuli and MATLAB code used for the preprocessing of the EEG data as well as R codes used for the analysis of behavioral data are available at Open Science Framework (OSF), https://osf.io/kx7bs/.

## References

[psyp13630-bib-0001] Arzouan, Y. , Goldstein, A. , & Faust, M. (2007). Brainwaves are stethoscopes: ERP correlates of novel metaphor comprehension. Brain Research, 1160, 69–81. 10.1016/j.brainres.2007.05.034 17597591

[psyp13630-bib-0002] Baldwin, C. L. , Roberts, D. M. , Barragan, D. , Lee, J. D. , Lerner, N. , & Higgins, J. S. (2017). Detecting and quantifying mind wandering during simulated driving. Frontiers in Human Neuroscience, 11, 406 10.3389/fnhum.2017.00406 28848414 PMC5550411

[psyp13630-bib-0067] Bambini, V. , Bertini, C. , Schaeken, W. , Stella, A. , & Di Russo, F. (2016). Disentangling Metaphor from Context: An ERP Study. Frontiers in Psychology, 7, 10.3389/fpsyg.2016.00559. PMC4853386 27199799

[psyp13630-bib-0003] Barr, D. J. , Levy, R. , Scheepers, C. , & Tily, H. J. (2013). Random effects structure for confirmatory hypothesis testing: Keep it maximal. Journal of Memory and Language, 68(3), 255–278. 10.1016/j.jml.2012.11.001 PMC3881361 24403724

[psyp13630-bib-0004] Bates, D. , Kliegl, R. , Vasishth, S. , & Baayen, R. H. (2015). Parsimonious mixed models. arXiv preprint:1506.04967 (stat.ME).

[psyp13630-bib-0005] Beaty, R. E. , Silvia, P. J. , & Benedek, M. (2017). Brain networks underlying novel metaphor production. Brain and Cognition, 111, 163–170. 10.1016/j.bandc.2016.12.004 28038366

[psyp13630-bib-0006] Beese, C. , Meyer, L. , Vassileiou, B. , & Friederici, A. D. (2017). Temporally and spatially distinct theta oscillations dissociate a language‐specific from a domain‐general processing mechanism across the age trajectory. Scientific Reports, 7(1). 10.1038/s41598-017-11632-z PMC5593879 28894235

[psyp13630-bib-0007] Benedek, M. , Beaty, R. , Jauk, E. , Koschutnig, K. , Fink, A. , Silvia, P. J. , … Neubauer, A. C. (2014). Creating metaphors: The neural basis of figurative language production. NeuroImage, 90, 99–106. 10.1016/j.neuroimage.2013.12.046 24384149 PMC3951481

[psyp13630-bib-0066] Blair, R. C. , & Karniski, W. (1993). An alternative method for significance testing of waveform difference potentials. Psychophysiology, 30(5), 518–524. 10.1111/j.1469-8986.1993.tb02075.x. 8416078

[psyp13630-bib-0008] Boden, M. A. (2004). The creative mind: Myths and mechanisms. Hove, UK: Psychology Press.

[psyp13630-bib-0009] Bornkessel‐Schlesewsky, I. , & Schlesewsky, M. (2008). An alternative perspective on “semantic P600” effects in language comprehension. Brain Research Reviews, 59(1), 55–73. 10.1016/j.brainresrev.2008.05.003 18617270

[psyp13630-bib-0010] Brouwer, H. , Fitz, H. , & Hoeks, J. (2012). Getting real about semantic illusions: Rethinking the functional role of the P600 in language comprehension. Brain Research, 1446, 127–143. 10.1016/j.brainres.2012.01.055 22361114

[psyp13630-bib-0012] Brysbaert, M. , New, B. , & Keuleers, E. (2012). Adding part‐of‐speech information to the SUBTLEX‐US word frequencies. Behavior Research Methods, 44(4), 991–997. 10.3758/s13428-012-0190-4 22396136

[psyp13630-bib-0013] Cacciari, C. , & Glucksberg, S. (1994). Understanding figurative language In Gernsbacher M. A. (Ed.), Handbook of psycholinguistics (pp. 447–477). San Diego, CA: Academic Press.

[psyp13630-bib-0014] Ceci, S. J. , & Liker, J. K. (1986). A day at the races: A study of IQ, expertise, and cognitive complexity. Journal of Experimental Psychology: General, 115(3), 255–266. 10.1037/0096-3445.115.3.255 2966233

[psyp13630-bib-0015] Chiesi, H. L. , Spilich, G. J. , & Voss, J. F. (1979). Acquisition of domain‐related information in relation to high and low domain knowledge. Journal of Verbal Learning and Verbal Behavior, 18(3), 257–273. 10.1016/S0022-5371(79)90146-4

[psyp13630-bib-0016] Chwilla, D. J. , Brown, C. M. , & Hagoort, P. (1995). The N400 as a function of the level of processing. Psychophysiology, 32(3), 274–285. 10.1111/j.1469-8986.1995.tb02956.x 7784536

[psyp13630-bib-0017] Cohen, J. (1988). Statistical power analysis for the behavioral sciences. New York, NY: Routledge Academic.

[psyp13630-bib-0018] Coulson, S. , & Van Petten, C. (2002). Conceptual integration and metaphor: An event‐related potential study. Memory & Cognition, 30(6), 958–968. 10.3758/BF03195780 12450098

[psyp13630-bib-0075] Coulson, S. , & Van Petten C. (2007). A special role for the right hemisphere in metaphor comprehension? Brain Research, 1146, 128–145. 10.1016/j.brainres.2007.03.008. 17433892

[psyp13630-bib-0019] De Grauwe, S. , Swain, A. , Holcomb, P. J. , Ditman, T. , & Kuperberg, G. R. (2010). Electrophysiological insights into the processing of nominal metaphors. Neuropsychologia, 48(7), 1965–1984. 10.1016/j.neuropsychologia.2010.03.017 20307557 PMC2907657

[psyp13630-bib-0020] Delorme, A. , & Makeig, S. (2004). EEGLAB: An open source toolbox for analysis of single‐trial EEG dynamics including independent component analysis. Journal of Neuroscience Methods, 134(1), 9–21. 10.1016/j.jneumeth.2003.10.009 15102499

[psyp13630-bib-0021] Ericsson, K. A. , & Kintsch, W. (1995). Long‐term working memory. Psychological Review, 102(2), 211–245. 10.1037/0033-295X.102.2.211 7740089

[psyp13630-bib-0022] Fauconnier, G. , & Turner, M. (1998). Conceptual integration networks. Cognitive Science, 22(2), 133–187. 10.1207/s15516709cog2202_1

[psyp13630-bib-0023] Filik, R. , Leuthold, H. , Wallington, K. , & Page, J. (2014). Testing theories of irony processing using eye‐tracking and ERPs. Journal of Experimental Psychology: Learning, Memory, and Cognition, 40(3), 811–828. 10.1037/a0035658 24548324

[psyp13630-bib-0024] Gibbs, R. W., Jr . (1994). Figurative thought and figurative language In Gernsbacher M. A. (Ed.), Handbook of psycholinguistics (pp. 411–446). Cambridge, MA: Academic Press.

[psyp13630-bib-0025] Giora, R. (2003). On our mind: Salience, context, and figurative language. Oxford, UK: Oxford University Press.

[psyp13630-bib-0026] Goldstein, A. , Arzouan, Y. , & Faust, M. (2012). Killing a novel metaphor and reviving a dead one: ERP correlates of metaphor conventionalization. Brain and Language, 123(2), 137–142. 10.1016/j.bandl.2012.09.008 23063676

[psyp13630-bib-0027] Groppe, D. M. , Urbach, T. P. , & Kutas, M. (2011). Mass univariate analysis of event‐related brain potentials/fields I: A critical tutorial review. Psychophysiology, 48(12), 1711–1725. 10.1111/j.1469-8986.2011.01273.x 21895683 PMC4060794

[psyp13630-bib-0072] Jaeger T. Florian (2008). Categorical data analysis: Away from ANOVAs (transformation or not) and towards logit mixed models. Journal of Memory and Language, 59(4), 434–446. 10.1016/j.jml.2007.11.007. 19884961 PMC2613284

[psyp13630-bib-0076] Jankowiak Katarzyna , Rataj Karolina , Naskręcki Ryszard (2017). To electrify bilingualism: Electrophysiological insights into bilingual metaphor comprehension. PLOS ONE, 12(4), e0175578 10.1371/journal.pone.0175578. 28414742 PMC5393611

[psyp13630-bib-0071] Jasper, H. (1958). Report of the committee on methods of clinical examination in electroencephalography. Electroencephalography and Clinical Neurophysiology, 10(2), 370–375. 10.1016/0013-4694(58)90053-1.

[psyp13630-bib-0028] Katz, A. N. (2005). Discourse and Sociocultural Factors in Understanding Nonliteral Language In Colston H. L. & Katz A. N. (Eds.), Figurative language comprehension: Social and cultural influences (pp. 183–207). Mahwah, NJ: Lawrence Erlbaum Associates Publishers.

[psyp13630-bib-0029] Katz, A. N. , & Ferretti, T. R. (2001). Moment‐by‐moment reading of proverbs in literal and nonliteral contexts. Metaphor and Symbol, 16(3–4), 193–221. 10.1080/10926488.2001.9678895

[psyp13630-bib-0030] Katz, A. N. , & Pexman, P. M. (1997). Interpreting figurative statements: Speaker occupation can change metaphor to irony. Metaphor and Symbol, 12(1), 19–41. 10.1207/s15327868ms1201_3

[psyp13630-bib-0031] Kazmerski, V. A. , Blasko, D. G. , & Dessalegn, B. G. (2003). ERP and behavioral evidence of individual differences in metaphor comprehension. Memory & Cognition, 31(5), 673–689. 10.3758/BF03196107 12956233

[psyp13630-bib-0032] Kutas, M. , & Federmeier, K. D. (2011). Thirty years and counting: Finding meaning in the N400 component of the event‐related brain potential (ERP). Annual Review of Psychology, 62, 621–647. 10.1146/annurev.psych.093008.131123 PMC4052444 20809790

[psyp13630-bib-0033] Kutas, M. , & Hillyard, S. A. (1980). Reading senseless sentences: Brain potentials reflect semantic incongruity. Science, 207(4427), 203–205. 10.1126/science.7350657 7350657

[psyp13630-bib-0073] Kuznetsova, A. , Brockhoff, P. B. , & Christensen, R. H. B. (2017). lmerTest Package: Tests in Linear Mixed Effects Models. Journal of Statistical Software, 82(13), 10.18637/jss.v082.i13.

[psyp13630-bib-0034] Lai, V. T. , Curran, T. , & Menn, L. (2009). Comprehending conventional and novel metaphors: An ERP study. Brain Research, 1284, 145–155. 10.1016/j.brainres.2009.05.088 19505446

[psyp13630-bib-0035] Lakens, D. (2013). Calculating and reporting effect sizes to facilitate cumulative science: A practical primer for t‐tests and ANOVAs. Frontiers in Psychology, 4, 863 10.3389/fpsyg.2013.00863 24324449 PMC3840331

[psyp13630-bib-0065] Lee, T.‐W. , Girolami M. , & Sejnowski T. J. (1999). Independent Component Analysis Using an Extended Infomax Algorithm for Mixed Subgaussian and Supergaussian Sources. Neural Computation, 11(2), 417–441. 10.1162/089976699300016719. 9950738

[psyp13630-bib-0037] Lopez‐Calderon, J. , & Luck, S. J. (2014). ERPLAB: An open‐source toolbox for the analysis of event‐related potentials. Frontiers in Human Neuroscience, 8 10.3389/fnhum.2014.00213 PMC3995046 24782741

[psyp13630-bib-0038] Luck, S. J. , & Gaspelin, N. (2017). How to get statistically significant effects in any ERP experiment (and why you shouldn't): How to get significant effects. Psychophysiology, 54(1), 146–157. 10.1111/psyp.12639 28000253 PMC5178877

[psyp13630-bib-0039] Matuschek, H. , Kliegl, R. , Vasishth, S. , Baayen, H. , & Bates, D. (2017). Balancing Type I error and power in linear mixed models. Journal of Memory and Language, 94, 305–315. 10.1016/j.jml.2017.01.001

[psyp13630-bib-0040] Mednick, S. (1962). The associative basis of the creative process. Psychological Review, 69(3), 220–232. 10.1037/h0048850 14472013

[psyp13630-bib-0069] Mullen, T. (2012). CleanLine EEGLAB plugin. San Diego, CA: Neuroimaging Informatics Toolsand Resources Clearinghouse (NITRC).

[psyp13630-bib-0074] Nieuwland, M. S. , & Van Berkum, J. J. A. (2006). When Peanuts Fall in Love: N400 Evidence for the Power of Discourse. Journal of Cognitive Neuroscience, 18(7), 1098–1111. 10.1162/jocn.2006.18.7.1098. 16839284

[psyp13630-bib-0041] Oldfield, R. C. (1971). The assessment and analysis of handedness: The Edinburgh inventory. Neuropsychologia, 9(1), 97–113. 10.1016/0028-3932(71)90067-4 5146491

[psyp13630-bib-0042] Olejnik, S. , & Algina, J. (2003). Generalized eta and omega squared statistics: Measures of effect size for some common research designs. Psychological Methods, 8(4), 434–447. 10.1037/1082-989X.8.4.434 14664681

[psyp13630-bib-0043] Pexman, P. M. (2008). It's fascinating research: The cognition of verbal irony. Current Directions in Psychological Science, 17(4), 286–290. 10.1111/j.1467-8721.2008.00591.x

[psyp13630-bib-0044] Pynte, J. , Besson, M. , Robichon, F.‐H. , & Poli, J. (1996). The time‐course of metaphor comprehension: An event‐related potential study. Brain and Language, 55(3), 293–316. 10.1006/brln.1996.0107 8954602

[psyp13630-bib-0045] Rabbitt, L. R. , Roberts, D. M. , McDonald, C. G. , & Peterson, M. S. (2017). Neural activity reveals perceptual grouping in working memory. International Journal of Psychophysiology, 113, 40–45. 10.1016/j.ijpsycho.2017.01.005 28088351

[psyp13630-bib-0046] Rataj, K. , Przekoracka‐Krawczyk, A. , & van der Lubbe, R. H. J. (2017). On understanding creative language: The late positive complex and novel metaphor comprehension. Brain Research, 1678, 231–244. 10.1016/j.brainres.2017.10.030 29107661

[psyp13630-bib-0068] R Core Team . (2020). R: A language and environment for statistical computing Vienna, Austria: R Foundation for Statistical Computing https://www.R‐project.org/

[psyp13630-bib-0047] Recht, D. R. , & Leslie, L. (1988). Effect of prior knowledge on good and poor readers' memory of text. Journal of Educational Psychology, 80(1), 16–20. 10.1037/0022-0663.80.1.16

[psyp13630-bib-0048] Ricks, T. R. , & Wiley, J. (2009). The influence of domain knowledge on the functional capacity of working memory. Journal of Memory and Language, 61(4), 519–537. 10.1016/j.jml.2009.07.007

[psyp13630-bib-0049] Rodd, J. M. , Cai, Z. G. , Betts, H. N. , Hanby, B. , Hutchinson, C. , & Adler, A. (2016). The impact of recent and long‐term experience on access to word meanings: Evidence from large‐scale internet‐based experiments. Journal of Memory and Language, 87(C), 16–37. 10.1016/j.jml.2015.10.006

[psyp13630-bib-0050] Rutter, B. , Kröger, S. , Hill, H. , Windmann, S. , Hermann, C. , & Abraham, A. (2012). Can clouds dance? Part 2: An ERP investigation of passive conceptual expansion. Brain and Cognition, 80(3), 301–310. 10.1016/j.bandc.2012.08.003 23137771

[psyp13630-bib-0051] Rutter, B. , Kröger, S. , Stark, R. , Schweckendiek, J. , Windmann, S. , Hermann, C. , & Abraham, A. (2012). Can clouds dance? Neural correlates of passive conceptual expansion using a metaphor processing task: Implications for creative cognition. Brain and Cognition, 78(2), 114–122. 10.1016/j.bandc.2011.11.002 22204876

[psyp13630-bib-0052] Schad, D. J. , Vasishth, S. , Hohenstein, S. , & Kliegl, R. (2020). How to capitalize on a priori contrasts in linear (mixed) models: A tutorial. Journal of Memory and Language, 110, 104038 10.1016/j.jml.2019.104038

[psyp13630-bib-0053] Schirmer, A. , & Gunter, T. C. (2017). The right touch: Stroking of CT‐innervated skin promotes vocal emotion processing. Cognitive, Affective, & Behavioral Neuroscience, 17(6), 1129–1140. 10.3758/s13415-017-0537-5 PMC5709431 28933047

[psyp13630-bib-0500] Schneider, S. , Rapp, A. M. , Haeußinger, F. B. , Ernst, L. H. , Hamm, F. , Fallgatter, A. J. , & Ehlis, A.‐C. (2014). Beyond the N400: Complementary access to early neural correlates of novel metaphor comprehension using combined electrophysiological and haemodynamic measurements. Cortex, 53, 45–59. 10.1016/j.cortex.2014.01.008 24566043

[psyp13630-bib-0054] Spilich, G. J. , Vesonder, G. T. , Chiesi, H. L. , & Voss, J. F. (1979). Text processing of domain‐related information for individuals with high and low domain knowledge. Journal of Verbal Learning and Verbal Behavior, 18(3), 275–290. 10.1016/S0022-5371(79)90155-5

[psyp13630-bib-0055] Sternberg, R. J. , & Lubart, T. I. (1996). Investing in creativity. American Psychologist, 51(7), 677–688. 10.1037/0003-066X.51.7.677

[psyp13630-bib-0056] Süß, H.‐M. , & Kretzschmar, A. (2018). Impact of cognitive abilities and prior knowledge on complex problem solving performance – Empirical results and a plea for ecologically valid microworlds. Frontiers in Psychology, 9 10.3389/fpsyg.2018.00626 PMC5952078 29867627

[psyp13630-bib-0057] Tang, X. , Qi, S. , Jia, X. , Wang, B. , & Ren, W. (2017). Comprehension of scientific metaphors: Complementary processes revealed by ERP. Journal of Neurolinguistics, 42, 12–22. 10.1016/j.jneuroling.2016.11.003

[psyp13630-bib-0058] Voss, J. F. , Vesonder, G. T. , & Spilich, G. J. (1980). Text generation and recall by high‐knowledge and low‐knowledge individuals. Journal of Verbal Learning and Verbal Behavior, 19(6), 651–667. 10.1016/S0022-5371(80)90343-6

[psyp13630-bib-0059] Ward, T. B. (1994). Structured imagination: The role of category structure in exemplar generation. Cognitive Psychology, 27(1), 1–40. 10.1006/cogp.1994.1010

[psyp13630-bib-0060] Ward, T. B. (2007). Creative cognition as a window on creativity. Methods, 42(1), 28–37. 10.1016/j.ymeth.2006.12.002 17434413

[psyp13630-bib-0061] Wiley, J. (1998). Expertise as mental set: The effects of domain knowledge in creative problem solving. Memory & Cognition, 26(4), 716–730. 10.3758/BF03211392 9701964

[psyp13630-bib-0062] Wiley, J. , George, T. , & Rayner, K. (2018). Baseball fans don't like lumpy batters: Influence of domain knowledge on the access of subordinate meanings. Quarterly Journal of Experimental Psychology, 71(1), 93–102. 10.1080/17470218.2016.1251470 27767384

[psyp13630-bib-0063] Winkler, I. , Debener, S. , Müller, K. R. , & Tangermann, M. (2015, August). On the influence of high‐pass filtering on ICA‐based artifact reduction in EEG‐ERP In 2015 37th Annual International Conference of the IEEE Engineering in Medicine and Biology Society (EMBC)(pp. 4101–4105). Milan, Italy: IEEE 10.1109/embc.2015.7319296 26737196

[psyp13630-bib-0064] Wu, W. , Keller, C. J. , Rogasch, N. C. , Longwell, P. , Shpigel, E. , Rolle, C. E. , & Etkin, A. (2018). ARTIST: A fully automated artifact rejection algorithm for single‐pulse TMS‐EEG data. Human Brain Mapping, 39(4), 1607–1625. 10.1002/hbm.23938 29331054 PMC6866546

